# Multi-Parameter Sensing Device to Detect Liquid Layers Using Long-Period Fiber Gratings

**DOI:** 10.3390/s18093094

**Published:** 2018-09-14

**Authors:** Zhihui Pan, Ying Huang, Hai Xiao

**Affiliations:** 1 School of Civil Engineering, Guangzhou University, Guangzhou 510006, China; ri13@163.com; 2 Department of Civil and Environmental Engineering, North Dakota State University, 1340 Administration Ave, Fargo, ND 58108, USA; 3 Department of Electrical and Computer Engineering, Clemson University, Clemson, SC 29634, USA; haix@clemson.edu

**Keywords:** layered liquid boundary, liquid level, long-period fiber grating sensor

## Abstract

Insoluble liquids show layers such as water and oil. The detection of the exact interface locations and the level changes for layered liquids are of paramount importance for chemistry purifications, liquid storage in reservoirs, oil transportation, and chemical engineering. However, accurately measuring liquid layers is challenging. This paper introduces a multi-parameter sensing device based on a long-period fiber grating (LPFG) sensor simultaneously detecting boundary and level changes of layered liquids. Laboratory experiments demonstrated that the sensor device would respond to the liquid interface change as a sharp and sudden resonant wavelength change, while it would show a gradual and steady resonant wavelength change to the level changes of layered liquids. The lab experiments also showed that the sensor device has a higher sensitivity when a higher LPFG cladding mode is used.

## 1. Introduction

For chemical processing, purification, storage, and transportation, it is critical to know whether unwanted liquids are present. If unwanted insoluble liquids exist, it becomes essential to locate the interface between the layered liquids and monitor the level change of each layer in real time. The measurement of liquid level change is widely applied. For instance, a fuel level sensor is commonly used in a car, truck or motorcycle to measure the gasoline level in the fuel tank. Sensor devices to detect level changes of liquid have been in place for decades. Various technologies are available including mechanical devices [1], electrical approaches [2,3,4], ultrasonic and acoustic technology [5].

A mechanical device estimates the liquid level through gauging the length of a wire on a floating ball or the gas pressure change inside an enclosed space [1]. Due to the implementation limitation of the mechanical device, it has a relatively low measurement accuracy. To measure more accurately, an electrical device can be used, such as magnetic, electrical conductivity, and capacitive sensing devices, through counting the changes of electrical-magnetic properties of the liquid [2,3,4]. These electrical devices can only be applied to liquids with significant changes of electrical or magnetic property towards liquid level changes. In addition, for some hazardous liquids, the use of these electrical devices may induce fire or explosion hazard. To have a safer field practice, an ultrasonic or acoustic sensor is an alternative to measure interface distance through finding the travel time of ultrasonic or acoustic waves [5]. However, due to the possible delays in sound wave measurements, an accurate level measurement requires high costs. Although the level sensors have been widely investigated and, to date, limited technologies can detect interfaces between layered insoluble liquids.

A fiber optic sensor, due to its unique advantages of compactness, immunity to electro-magnetic noise and moisture, no fire or explosion hazard, and long life cycle, can be a potential candidate for detecting layers of liquids. Various optic fiber sensors have been investigated for liquid level sensing such as intensity-based sensors [6], fiber Bragg grating [7,8], and long-period fiber gratings (LPFG). Among these fiber optic sensors, intensity-based sensors and fiber Bragg gratings are usually applied as on/off sensor for level warning. LPFG couples the core mode to a co-propagating cladding mode which decays rapidly as they propagate along the fiber axis from the scattering losses at the cladding-air interface in the fiber [9]. One cladding mode experiences total internal reflection at the interface between the cladding and the surrounding environment, resulting in a dependence of the effective index of the cladding mode on the surroundings environments.

The LPFG sensors have been widely applied in chemical, temperature and strain sensing [10,11,12]. Due to high sensitivity to environmental changes, LPFGs can potentially be a continuous liquid level sensor. Khaliq et al. [13] first applied LPFGs to roughly demonstrate oil level detection. The sensor showed a large linear range with sensitivity of 4.8% change in transmission per millimeter. Grice et al. [14] repeated the experiment and found that the level change of the liquid with a specific refractive index would induce both a shift in wavelength and a change in the attenuation level of the selected loss band. Hu et al. [15] and Xue et al. [16] formed LPFGs into interferometer sensors to detect liquid level changes. Recently, LPFG sensors also have been investigated for multi-parameter sensing in liquids. In 2012, Wang et al. [17] used LPFG sensor to simultaneously detect liquid level and flow velocity. Later, Huang et al. [18] developed LPFG sensors for simultaneous measurements of liquid level and refractive index. Yu et al. [19] combined LPFG and tilted FBG for two-parameter sensing in liquids. More recently, Jiang et al. [20] has advanced the LPFG sensor to simultaneously measure liquid refractive index and temperature.

Although LPFG sensors have been investigated for detecting liquid levels and multiple parameter sensing in liquids, limited research is done for liquid layer detection. The common approaches used for detecting the interfaces among various liquids are still highly dependent on visible examination or electrical capacitive sensors [21], which is inaccurate and in most cases not safe to operate. Although recently the authors introduced LPFGs for layered liquid detection [22], limited tests were performed to show its reliability. To meet this challenge, this study (1) systematically investigated the operational principle and feasibility of the LPFG-based sensing device for boundary detection between layered liquids, (2) advanced the measurements to multiple parameter detection of layered liquids with both the interfaces and the level changes of layered liquids, (3) validated the multi-parameter sensing through well-designed laboratory tests; and (4) performed sensitivity study on the sensor device for the rod diameter, material, and LPFG cladding modes selection. The paper is organized as follows: Section 2 discusses the sensor operational principle and device design; Section 3 explains the experimental setup; Section 4 conducts experiments to validate feasibility and effectiveness of multi-parameter sensing; Section 5 performs a sensitivity study of the sensor device through laboratory experiments; and Section 6 concludes the study and lay out its potential future work.

## 2. Operational Principles and Device Design

### 2.1. Principles of Operation

**Figure 1:**
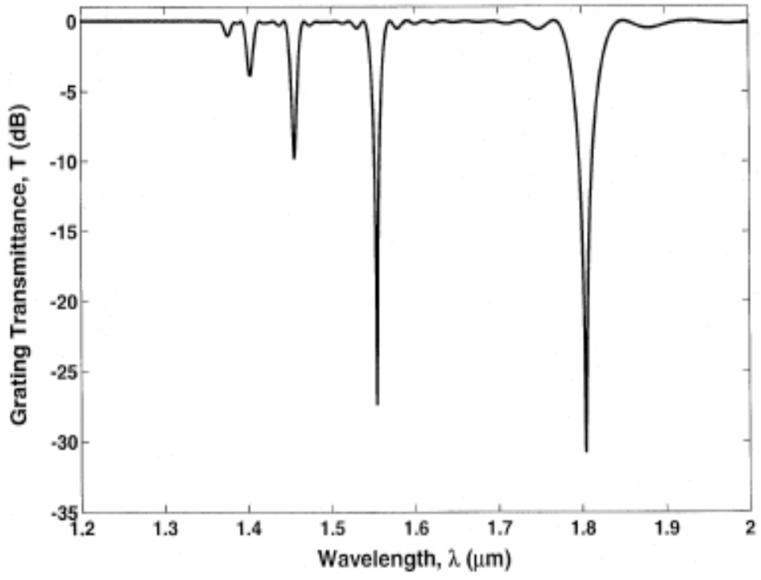
Representative LPFG transmission response.

The LPFGs used in this paper were fabricated by CO_2_ laser irradiation technique following reference [23]. The fiber used to fabricate the LPFG is single mode optical fiber (supplied by Corning). The LPFG has a sensor length of approximately 30–32 mm with 80–90 points of CO_2_ laser radiation and a period of 0.363–0.375 mm. Figure 1 shows a typical spectrum of one CO_2_ laser-induced LPFG with five different cladding modes (LP_01_–LP_05_). Each valley in Figure 1 corresponds to one cladding mode. The resonant wavelength (*λ_res_*) of a LPFG sensor can be expressed as a linear function of its grating period (Λ) and effective refractive indices of the core ($n_{eff,\;co}$) and the cladding mode LP_0*m*_ ($n_{eff,\;cl,\;m}$) as follows [9]:

(1) $$\lambda_{res}=(n_{eff,\;co}-n_{eff,\;cl,\;m})\Lambda$$

In Equation (1), the effective refractive indices of the core, $n_{eff,\;co}$, is a constant which will not be affected by changing surrounding medium. The grating period, Λ, although responds to physical changes such as temperature and stress on the fiber, it usually does not change with surrounding medium changes. However, the effective refractive index of the cladding mode LP_0*m*_, $n_{eff,\;cl,\;m}$, is highly dependent on the refractive index of the surrounding mediums, *n_s_*. A change in *n_s_* will bring in a corresponding change of $n_{eff,\;cl,\;m}$ in Equation (1), leading to a shift of the resonant wavelength of the cladding modes, *λ_res_*. The LPFG resonant wavelength sensitivity on *n_s_* can be obtained as [24]:

(2) $$\frac{d\lambda_{res}}{dn_{s}}=\lambda_{res}\cdot \gamma \cdot \Gamma_{s}$$

where, *γ* describe the waveguide dispersion which is positive for lower cladding modes and negative for higher cladding modes and the turning point of *γ* is highly dependent on the resonant wavelength range [24]. To avoid turning points and have a high sensitivity on the refractive index changes of surrounding mediums, an appropriate cladding mode needs to be selected. Section 5.3 performs a detail experimental investigation on influence of LPFG cladding modes on d*λ_res_*/d*n_s_* and selects the best cladding modes for the targeted application. Γ*_s_* expresses the dependence of the grating to the surrounding refractive index and is defined by reference [24], which is negative for all the cladding modes.

The LPFG surrounding medium changes from one liquid to another along any location during the length of the sensing unit will introduce a sudden change of Γ*_s_* in Equation (2), resulting in a change in the sensor sensitivity, d*λ_res_*/d*n_s_*. Thus, a sudden change in the sensitivity of the sensor unit, which is the slope of the level measurements, indicates the occurrence of boundaries in layered insoluble liquids. The interfaces between layered liquids locate at wherever the change of sensitivity change occurs along the sensor unit. From Equation (2), it shows that the resolution of interface between liquids can be one grating period, which is in between 0.363 mm to 0.375 mm.

Equation (2) also shows that if the properties of layered liquids remain unchanged after the occurrence of insolvable liquids, the Γ*_s_* will remain as a constant and the sensitivity of the LPFG will remain unchanged. Then, Equation (1) with sensitivity from Equation (2) can be used to continuously monitor level changes until the liquids emerging the entire sensor unit. For more details of the LPFG sensing principle about liquid level changes, it can be referred to [24]. Therefore, based on Equations (1) and (2), a sensor device based on LPFG sensing unit can potentially be used to detect multiple parameters of layered liquids, including the interface locations and the level changes of each layer. From Equations (1) and (2), it can be seen that if a higher resolution for liquid level measurement is desired, a smaller LPFG period, Λ, may yield a better result [24]. However, a smaller period will result in a short LPFG length leading to a smaller measurement range. In this study, all the LPFG sensors used share the same LPFG period and length to exclude the impacts from LPFG fabrication, which will be investigated in future.

### 2.2. Sensor Device Design

**Figure 2:**
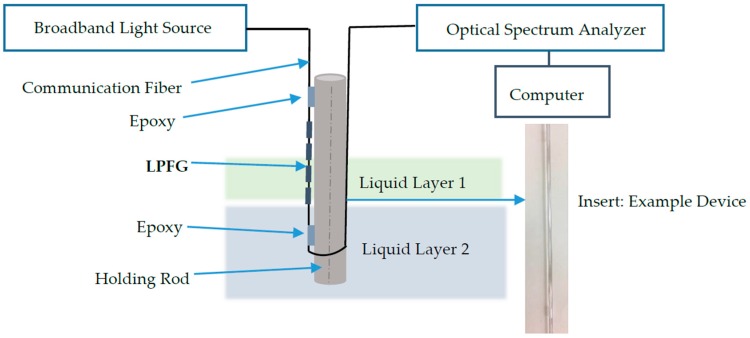
Schematic of the sensor device.

To measure multiple parameters for layered liquids as described in Section 2.1, the sensor device is expected to be robust, mobile, and ease to use. However, a single LPFG sensing unit made up of glass fiber with a diameter of 125 µm is too weak to be a probe for any possible lab and field detection. This paper designs a sensor device based on LPFGs to perform robust layered liquid measurements. Figure 2 shows a schematic view of the sensor device. The sensor device consists of two components: a LPFG sensing unit and a holding rod. The LPFG sensing unit is attached on the surface of the holding rod using epoxy on the both ends of the sensing unit. To avoid the phenomenon of capillarity, the epoxy on both ends of the sensing unit has a thickness of more than 2 mm. The material of the holding rod can be glass or metal. The diameter of the holding rod cannot be too small to avoid capillarity effect on measurements. A detail experimental investigation on the sensitivity study of the sensor device on the material and diameter of the holding rod will be discussed in Section 5.1 and Section 5.2.

As also shown in Figure 2, to measure the interface in between layered liquids and the level changes simultaneously, light from the broadband light source will go through the transmission fiber and the LPFG sensing unit which is attached on the holding rod. The light will then be picked up and recorded continuously using an optical spectrum analyzer. The optical spectrum analyzer will be connected to a personal computer to perform data analysis and plot the monitored data in user-friendly view.

**Figure 3:**
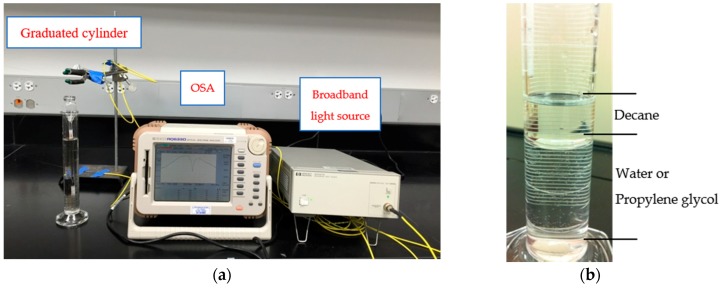
(**a**) Experimental setup; (**b**) Photo for the layered liquids.

To further protect the sensing unit from external damages during transportation of the sensor device, a protection plastic or glass tube with an open end and larger diameter than the holding rod can be applied to host the holding rod. The open end of the tube allows layered liquids to come into the tube for measurement. An example of the protection tube can be seen in Figure 3a. For practical application, the protected sensor device will be mounted or attached on the storage tanks or containers at the locations where potential layered liquids want to be monitored. Thus, if any interfaces of layered liquids occur or any changes in liquid level during the length of the sensing unit, spectrum changed of the transmitted light, which will be recorded by the optical spectrum analyzer, plotted and showed in the personal computer to the interested users.

## 3. Experiment Setup

Laboratory experiments were conducted to validate the developed sensor device for multi-parameter measurements of layered liquids on liquid interface location and level changes. More importantly, experiments were also performed to investigate the influences of several key factors on the sensitivity of the sensor device, including the materials and diameter of the holding rod, and the cladding mode of the LPFG sensors for different needs in practical applications.

Three layered liquids were used for laboratory validation in this paper including water, decane, and propylene glycol. All these three liquids are colorless and odorless. Decane is an alkane hydrocarbon with the chemical formula CH_3_(CH_2_)_8_CH_3_, which is one of the components in gasoline (petroleum). Decane is nonpolar and will not dissolve in polar liquids such as water. It has a refractive index of 1.41, which is larger than water of 1.33 in refractive index [25]. Propylene glycol is a synthetic organic compound with the chemical formula C_3_H_8_O_2_, which is primarily used in the production of polymers but also sees use in food processing [26]. Propylene glycol is miscible with water but insoluble with decane. It has a refractive index of 1.43, which is larger than that of decane (1.41). It is known based on Equations (1) and (2) that temperature may introduce significant measurement variances which is not related to any liquid layer or level measurements. Thus, in this study, all the laboratory tests were performed in a controlled temperature environment at room temperature (22 °C). For practical applications, a temperature compensation LPFG sensor is recommended to be installed parallel to the LPFG unit on the sensor device to eliminate the temperature effects on the sensor device.

Figure 3a shows the experimental setup to detect the layered liquids using the developed sensor device. The developed sensor device was protected inside a glass tube with a larger diameter. The protected sensor device was then placed inside a graduated cylinder. To perform a systematic study on the detection of the liquid layer interface for various insolvable liquids and understand the sensitivity of the device towards various parameters which may influence the detection, ten sets of laboratory experiments had been performed in the controlled room temperature environments as shown in Table 1. Among the ten experiments, three sets of laboratory tests were performed on only one liquid as surrounding environment including Experiment #1 for water only, #2 for decane only, and #3 for propylene glycol only. In addition, Experiment #1 was compared with numerical simulations to validate the effectiveness of the experimental setup. Two more sets of experiments were conducted on different combinations of liquids with Experiment #4 for water and decane and Experiment #5 for propylene glycol and decane. Experiments #1 to #5 used the same rod material (glass) and size (outer diameter of 3 mm), and the same LPFG cladding mode of LP_07_.

In addition to the laboratory validation experiments, sensitivity tests were also performed on device including rod materials (Experiment #6), rod sizes (Experiment #7), and LPFG cladding modes (Experiment #8 and #9). Two materials were compared including glass (Experiment #4) and metal (Experiment #6) using the same outer diameter of 3 mm and LPFG cladding mode of LP_07_. Two sizes of rod including outer diameter of 3 mm (Experiment #4) and 2 mm (Experiment #7) were compared using the glass rod and same LPFG cladding mode of LP_07_. Three LPFG cladding modes were analyzed including of LP_06_ (Experiment #8), LP_07_ (Experiment #4), and of LP_08_ (Experiment #9) using glass rod with outer diameter of 3 mm. All the liquid was gradually added into the graduated cylinder as shown in Figure 3b. Since the resolution of the LPFG for liquid interface changes is one LPFG grating period, which follows in between 0.363 mm to 0.375 mm in this study, a gradual change of 1 mm was used in laboratory experiments to ensure the device can detect the liquid interface accurately. The LPFG sensor was connected in series to a broadband light source ranging from 1520 to 1620 nm and to an optical spectrum analyzer (OSA), followed by a personal computer. The spectrum changes of the LPFG sensor were recorded using the OSA and personal computer throughout all the experiments.

## 4. Multi-Parameter Sensing Experimental Results and Discussions

### 4.1. Liquid Level Measurements for One Liquid Only (Experiment #1 to #3)

**Figure 4:**
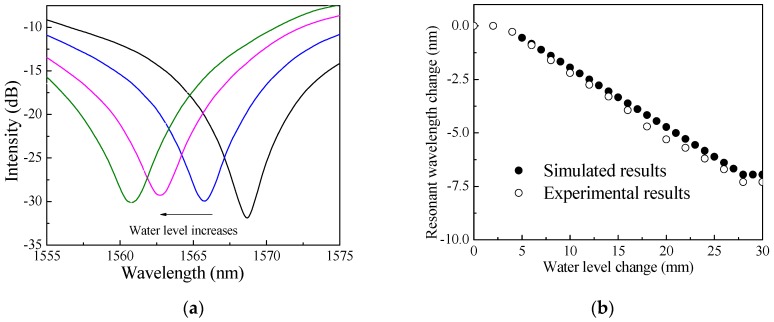
(**a**) Transmission spectrum; (**b**) Resonant wavelength changes of LP_07_ to water level changes.

In Experiment #1 to #3, water (*n_s_* = 1.33), decane (*n_s_* = 1.41), and propylene glycol (*n_s_* = 1.43) was gradually added into the graduated cylinder, respectively as shown in Figure 3a. Figure 4a shows the example spectrum changes of the LPFG sensing unit as the water level increases. In addition to laboratory experiments, numerical simulation was also performed for the cladding mode of LP_07_ following the procedures in reference [18]. Figure 4b compares the experimental resonant wavelength changes of the cladding mode of LP_07_ to simulations for a water level from 0 to 30 mm (full length of the grating) according to Equation (1). Figure 4b shows that the experimental and simulated results on the resonant wavelength changes agree with each other very well. Overall, the water level sensitivity of the grating from the test data between 5 mm and 27 mm is −0.220 nm/mm for the cladding mode of LP_07_. The simulation yielded a water level sensitivity of −0.222 nm/mm for LP_07_. The difference between the experimental data and simulations is likely due to the human error of variance for uneven water level addition during the tests.

**Figure 5:**
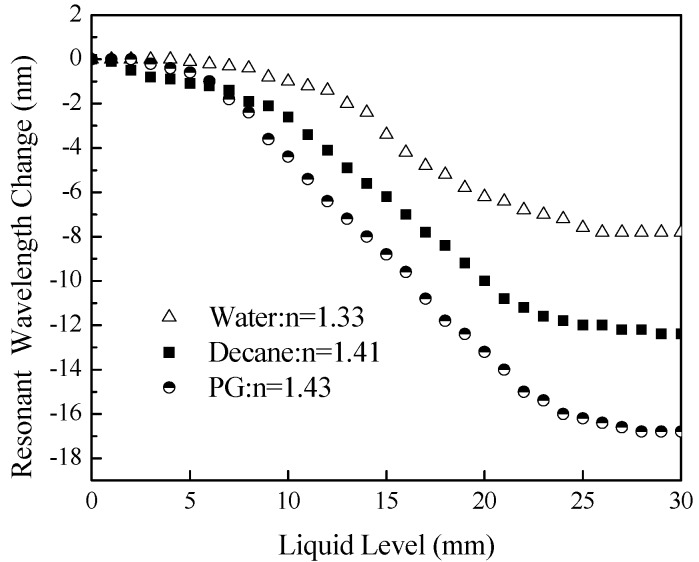
Resonant wavelength changes of LP_07_ to the change of levels in water, decane, and propylene glycol (PG).

Figure 5 shows the comparison resonant wavelength changes of the cladding mode LP_07_ to the change of levels in water, decane, and propylene glycol for Experiments #1–3. The slope changes of the LPFG resonant wavelength changes can distinguish different types of liquids and the gradual resonant wavelength changes can provide information on liquid level changes.

### 4.2. Layered Liquid Interface and Level Detection (Experiment #4 to #5)

**Figure 6:**
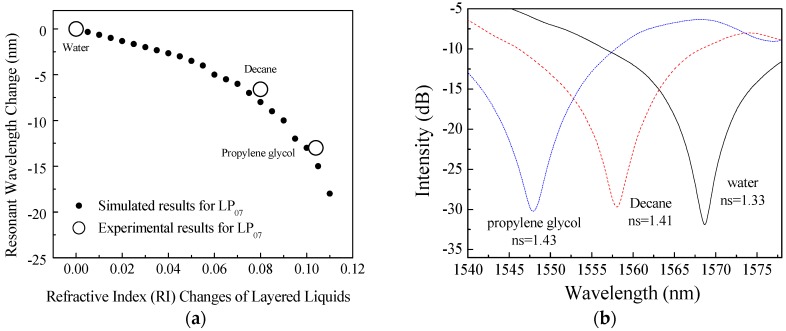
(**a**) Experimental and simulated resonant wavelength changes of LP_07_ in responding to changes in liquid interfaces; (**b**) Spectrum changes with liquid interface changes.

Before Experiment #4 to #5, simulations and experiments were performed on the LPFG cladding mode of LP_07_ with a surrounding liquid of refractive index changing from 1.33 to 1.44 by an interval of 0.005. The simulation was performed following the procedure in reference [27]. Figure 6a shows the corresponding simulated resonant wavelength changes to the change of the surrounding liquids referred to water (*n* = 1.33) as the base liquid, according to Equations (1) and (2). The relation between the resonant wavelength changes and the refractive index changes of the surrounding layered liquids is nonlinear, particularly towards a large index value. In the laboratory experiments, the three liquids used include the liquids used in Experiments #4 and #5, which are water (*n_s_* = 1.33), decane (*n_s_* = 1.41) and propylene glycol (PG) (*n_s_* = 1.43). The unfilled circles in Figure 6a represent the resonant wavelength changes measured from the LPFG sensor device for the cladding modes LP07 and Figure 6b shows the recorded spectrums for the interface changes between three different layered liquids. It can be seen from Figure 6a that the simulated results are in good agreement with the experimental data. The simulation predicted that a change of −7.500 nm will occur the surrounding liquid changing from water to Decane, and the experiment showed to be −6.700 nm. The slight difference between them is likely attributable to the potential variation of concentration in the tested liquids.

**Figure 7:**
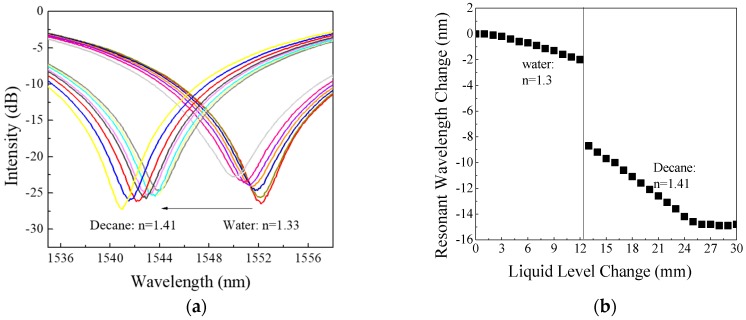
(**a**) Transmission spectrum; (**b**) Resonant wavelength changes of LP_07_ vs. simultaneous liquid interface and level changes (water and decane).

With the expectation of sudden resonant wavelength changes when the liquid boundary of layers presented during the length of LPFG, Experiment #4 was conducted to validate the detection of boundary between decane and water. Figure 7a shows the recorded transmission spectrum changes of Experiment #4 with boundary changes between water and decane for the cladding mode LP_07_ and Figure 7b shows its resonant wavelength changes with adding water and decane. The interface in between the two different layered liquids is clearly identified at the location of 12.5 mm away from the start point of the LPFG sensing unit with a sudden center wavelength change of 6.6 nm on the boundary between the water and Decane. When compared to Figure 6a and Figure 5, the experiments showed great consistence with multiple experiments. Before the decane was added, the center wavelength of LPFG shifts to left with a sloop of −0.220 nm/mm. After the liquid layer interface presents, the resonant wavelength of the LPFG continued shifting to left as the level of decane increases at a rate of −0.504 nm/mm. Figure 7a,b clearly show that the developed sensor device can detect the interfaces between layered liquids and at the same time the level changes of the associated layered liquids.

**Figure 8:**
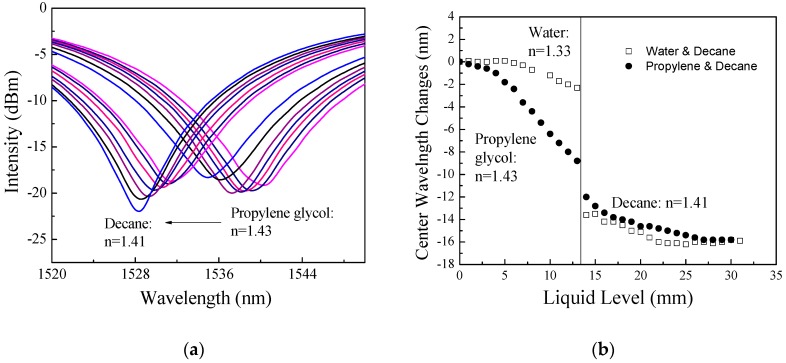
(**a**) Spectrum changes vs. simultaneous liquid interface and level changes (Experiment #5); (**b**) Resonant wavelength changes of LP07 vs. simultaneous liquid interface and level changes (Experiments #4 and #5).

Figure 8a shows the recorded transmission spectrum changes of Experiment #5 with boundary changes between PG and decane for the cladding mode LP_07_ and Figure 8b shows the resonant wavelength changes for Experiments #4 and #5 with adding water followed by decane and adding propylene followed by decane. Figure 8b shows that a bigger difference in refractive index as water (*n* = 1.33) and decane (*n* = 1.41) would introduce a bigger resonant wavelength change when compared to smaller difference in refractive index as PG (*n* = 1.43) and decane (*n* = 1.41).

## 5. Device Sensitivity Study

### 5.1. Influence from Rod Materials (Experiment #4 and #6)

**Figure 9:**
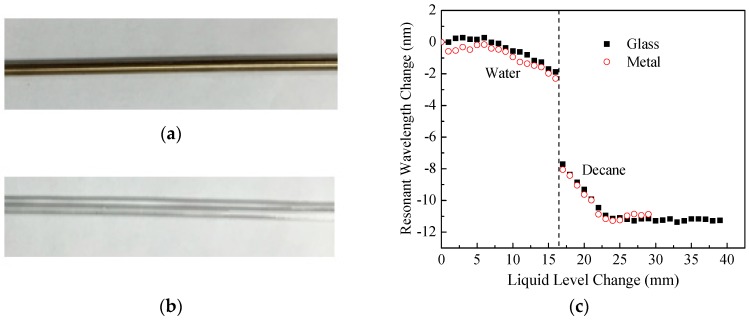
(**a**) Metal holding rod of 3 mm; (**b**) Glass holding rod of 3 mm; (**c**) Resonant wavelength changes of the LP_07_ vs. layered liquid changes for two holding rods with different materials: metal and glass in layered liquids of water and decane.

To investigate the influence of different holding rod materials on the sensitivity of the developed sensor device, two different rod materials were tested in lab, including metal and glass material as shown in Figure 9a,b for the photos of the two tested rods. Both rods had same outer diameter of 3 mm. Figure 9c shows the experimental results for measuring level changes and interface of layered liquids, water and decane. The liquid level sensitivity of the LPFG sensing unit did not change significantly with the use of the two different materials of the holding rod. On the glass rod, the sensor had a level sensitivity for water of −0.222 nm/mm and −0.505 nm/mm for decane. On metal rod, the sensor had a level sensitivity of −0.205 nm/mm for water and −0.504 nm/mm for decane, respectively. The materials did affect a little on the actual value change of the interface detection. Glass rod showed 6.66 nm changes between the interface of water and decane, and metal rod had 6.76 nm changes. The 0.1 nm difference between the two different materials of the holding rods can be attributed to the variance of interface locations when performing experiments two different times. Thus, these experiments provide confidence in ignoring the influence from materials of the holding rods. For practical applications, best available and most cost-effective holding tube material can be used. However, laboratory calibration is recommended before any field applications of the sensor device.

### 5.2. Influence from Rod Diameters (Experiment #4 and #7)

**Figure 10:**
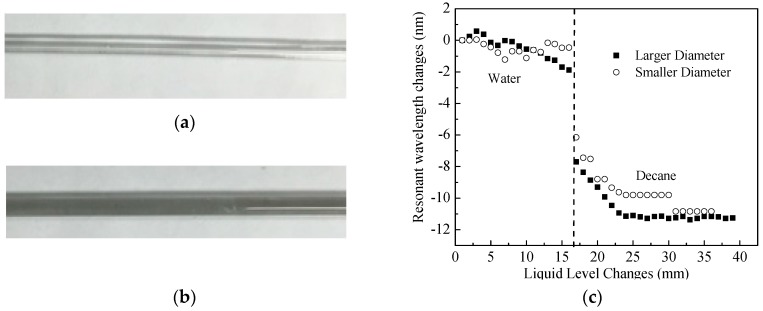
(**a**) Glass holding rod with outer diameter of 2 mm; (**b**) Glass holding rod with outer diameter of 3 mm; (**c**) Resonant wavelength changes of the LP_07_ vs. layered liquid changes for two holding rods with two different sizes in layered liquids of water and decane.

The influence of rod diameter on the sensitivity of the developed sensor device was also tested in laboratory by comparing the sensing responses of two different diameters of the glass holding rods. Figure 10a,b show the photos of the two tested glass rods with different diameters of 2 mm and 3 mm. Figure 10c shows the experimental results for measuring level changes and interface of layered liquids for water and decane. For the two different diameters of the holding rod, the sensor device performed well for liquid interface measurements with similar center wavelength changes of 5.68 nm and 5.82 nm, respectively. However, Figure 10c clearly shows that the diameter of the holding rod influences the accuracy of the liquid level measurement significantly. The sensor device with smaller diameter in glass holding rod of 2 mm outer diameter failed to measure the level changes in water although it behaved well in decane. The phenomenon can be explained by the capillarity effect. A smaller diameter of a holding rod may introduce significant capillarity effect, which the thickness of a small epoxy layer cannot overcome. Therefore, for practical applications, the diameter of a holding rod of the sensor device is recommended to be at least larger than 3 mm according to our experimental data. In addition, laboratory calibration is required before any field applications to ensure an effective size of the holding rod. If the sensor device changes its size of the hosting rod, it cannot be assumed to have the same sensor sensitivity without laboratory calibration.

### 5.3. Influence from LPFG Cladding Modes (Experiment #4, #8 and #9)

In Section 2.1, Equation (2) predicts that a higher cladding mode of LPFG sensor can result in a larger Γ*_s_*, leading to a higher sensitivity to measure the surrounding medium changes such as liquid interface or level changes. From previous study [18], although the resonant wavelength changes with liquid nonlinearly, linear approximation can be used to estimate the liquid level changes. More details about the nonlinearity of the liquid level to the resonant wavelength change refer to [18]. In addition, if the cladding mode is too high, it may show a turning point in spectrum, which is undesired for this application [28]. To investigate the influence of LPFG cladding modes on sensitivity of the developed sensor device, two other LPFG cladding modes, LP_06_ and LP_08_, in addition to the LP_07_ as shown in the previous sections from Figure 6, Figure 7 and Figure 8, have been investigated here. All the three cladding modes (LP_06_, LP_07_, and LP_08_) are visible in a 400 nm wavelength range. For these three cladding modes, no dual resonance is expected to present with the surrounding refractive index changing from 1.0 to 1.46 [28], which is the common range for liquids.

**Figure 11:**
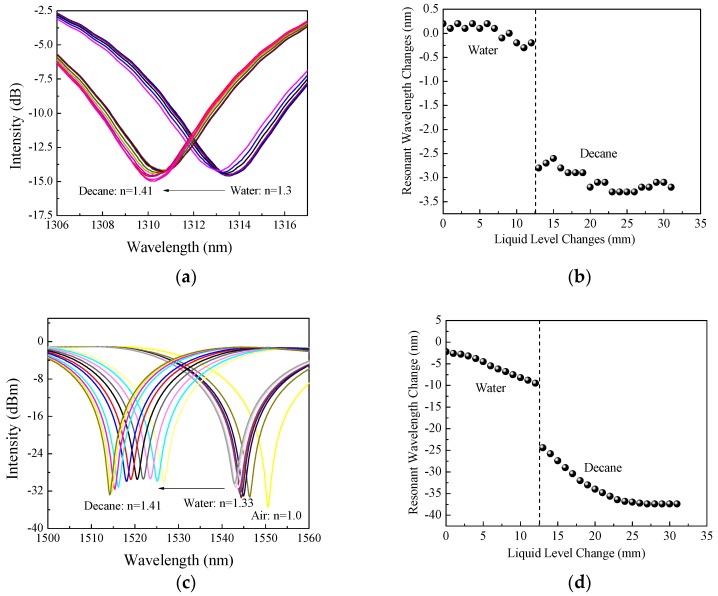
(**a**) Spectrum changes of LP_06_; (**b**) Resonant wavelength changes of the LP_06_; (**c**) Spectrum changes of LP_08_; (**d**) Resonant wavelength changes of the LP_08_ with layered liquid interface and level changes (water and decane).

Figure 11a shows the transmission spectrum changes of LP_06_ with adding water followed by decane and Figure 11b shows its resonant wavelength changes. A similar phenomenon as LP_07_ (shown in Figure 7) was observed for LP_06_; however, lower sensitivity is observed for LP_06_ for both liquid interface and level measurements. For water, the resonant wavelength of the LP_06_ was gradually shifting to the left with a sloop of −0.041 nm/mm. For decane, the level sensitivity LP_06_ was −0.059 nm/mm. In between water and decane, a sudden resonant wavelength shift occurred with a change of 2.5 nm. The boundary between water and decane was clearly detected by the LP_06_ cladding mode also at 12.5 mm away from the beginning of the LPFG starting point. Comparing Figure 7b to Figure 11b, it can be clearly seen that the sensor sensitivity of the cladding mode LP_06_ was five times smaller than the cladding mode of LP_07_ for both liquid interface and level measurements.

For the cladding mode LP_08_, the spectrum changes with the liquid level and interface changes could be found in Figure 11c and the corresponding resonant wavelength changes could be found in Figure 11d. From the experimental results, it can be seen that a big change of resonant wavelength occurred for LP_08_ when decane was added into water. For water, the LP_08_ had a level sensitivity of −0.643 nm/mm and for decane, it has a level sensitivity of −1.154 nm/mm. The center wavelength shift for LP_08_ at the interface in between water and the decane is 16.2 nm. Comparing Figure 7b to Figure 11d, the cladding mode of LP_08_ is much more sensitive with at least two times higher than that of the cladding mode LP_07_. No peak split occurred along the experiments. The interface of the water and decane was also located to be at 12.5 mm away from the LPFG starting point.

The comparison between Figure 6 and Figure 9 clearly indicates that a higher cladding mode of a LPFG sensing unit will have a higher sensitivity for detecting the layered liquids. The LP_08_ is two times more sensitive than the LP_07_, and the LP_07_ is five times more sensitive than the LP_06_. For practical applications, it is recommended to use the highest cladding modes with no peak split in the range of liquids to be measured to achieve the highest sensitivity of measurements.

## 6. Conclusions and Future Work

In this paper, a sensor device based on LPFG was developed to detect simultaneous changes in the interface and level of insoluble layered liquids. Based on the findings from this paper, the following conclusions can be drawn:

(a) Operational principles of the sensor device show that a constant slope will be present for level changes and a sudden change in the slope of the level measurements is expected for the occurrence of boundaries in layered insoluble liquids.
(b) The laboratory experiments clearly validated that the developed sensor device could detec the interface and level changes of layered liquid using the example of water, decane, and propylene glycol.
(c) Experimental study also showed that the materials of the holding rods of the LPFG sensor on the sensor device had very little influence on the sensor’s sensitivity; however, the diameter of the holding rod did influence the accuracy of the device which will require a calibration before the sensor’s practical application.
(d) The selection of cladding modes of the LPFG will also affect significantly the sensitivity of the sensor device. A cladding mode of LP_08_ was tested to be two times more sensitive when compared to LP_07_, and more than ten times more sensitive than LP_06_.

In the future, more investigation will be performed to identify the liquid chemical components and apply the developed sensor device for field applications. With the advantages of high measurement accuracy and great durability, the developed optical fiber sensing device could be applied for layered liquid detection in various applications such as fuel storage systems, chemical processing, and aerospace engineering. However, since the sensitive range of the developed device is limited by the length of the LPFG unit, if the boundary of the layered liquids is not located within the length of the LPFG sensor unit, it may not be able to detect the boundary. For practical applications which require a larger detection range, multiple LPFG units as a series or long-gauged LPFG sensor unit is suggested to obtain a potential larger measurement range, which will be studied in future.

## Figures and Tables

**Table 1:** Laboratory Test Matrix.

Experiment No.	Liquids Used	Rod Material, Rod Size, and LPFG Cladding Modes
#1	Water Only	Glass rod with 3 mm outer diameter, LP_07_
# 2	Decane Only	Glass rod with 3 mm outer diameter, LP_07_
# 3	Propylene Glycol Only	Glass rod with 3 mm outer diameter, LP_07_
# 4	Water + Decane	Glass rod with 3 mm outer diameter, LP_07_
# 5	Propylene glycol + Decane	Glass rod with 3 mm outer diameter, LP_07_
# 6	Water + Decane	Metal rod with 3 mm outer diameter, LP_07_
# 7	Water + Decane	Glass rod with 2 mm outer diameter, LP_07_
# 8	Water + Decane	Glass rod with 3 mm outer diameter, LP_06_
# 9	Water + Decane	Glass rod with 3 mm outer diameter, LP_08_

## References

[1] Beutel T., Ferreira N., Leester-Schädel M., Büttgenbach S. Robust pressure sensor for measurements in boundary layers of liquid fluids with medium total pressures. Proceedings of the International Society for Optical Engineering.

[2] Infineon Technologies AG (2009). Liquid Level Sensing Measuring Liquid Levels Using Hall Effect Sensors. https://www.infineon.com/dgdl/AppNote_Liquid_Level_Sensing_Rev.1.0.pdf?fileId=db3a30432313ff5e0123a385f3b2262d.

[3] Makanjuola N.T., Shoewu O.O., Akinyemi L.A., Ajasa A.A. (2015). Design and development of microcontroller based liquid level detector with graphical output. Pac. J. Sci. Technol..

[4] Kumar B., Rajita G., Mandal N. (2014). A review on capacitive-type sensor for measurement of height of liquid level. Meas. Control.

[5] Meribout M., Habli M., Al-Naamany A., Al-Busaidi K. A new ultrasonic-based device for accurate measurement of oil, emulsion, and water levels in oil tanks. Proceedings of the 21st IEEE Instrumentation and Measurement Technology Conference.

[6] Wang A., Gunther M.F., Murphy K.A., Claus R.O. (1992). Fiber-optic liquid-level sensor. Sens. Actuators A.

[7] Guo T., Zhao Q., Duo Q., Zhang H., Xue L., Huang G., Dong X. (2005). Temperature-insensitive fiber bragg grating liquid-level sensor based on bending cantilever beam. IEEE Photonics Technol. Lett..

[8] Wang D., Cao M., Li C., Li D., Chen Y., Xu X., Xu J., Li Y., Wan Z., Wang B. (2011). Fiber bragg grating liquid level sensor with double pressure and temperature sensitivities. Procedia Eng..

[9] Bhatia V. (1996). Optical fiber long period grating sensors. Opt. Lett..

[10] Ye X.W., Su Y.H., Han J.P. (2014). Structural health monitoring of civil infrastructure using optical fiber sensing technology: A comprehensive review. Sci. World J..

[11] Zheng S., Zhu Y., Krishnaswamy S. (2013). Fiber humidity sensors with high sensitivity and selectivity based on interior nanofilm-coated photonic crystal fiber long-period gratings. Sens. Actuators B.

[12] Tian F., He Z., Du H. (2012). Numerical and experimental investigation of long-period gratings in photonic crystal fiber for refractive index sensing of gas media. Opt. Lett..

[13] Khaliq S., James S.W., Tatam R.P. (2001). Fiber-optic liquid-level sensor using a long-period grating. Opt. Lett..

[14] Grice S., Zhang W., Sugden K., Bennion I. Liquid level sensor utilizing a long period fiber grating. Proceedings of the International Society for Optical Engineering.

[15] Fu H., Shu X., Zhang A., Liu W., Lin Z., He S., Bennion I. (2011). Implementation and characterization of liquid-level sensor based on a long-period fiber grating Mach–Zehnder interferometer. IEEE Sens. J..

[16] Xue H., Xu Z., Chen H., Yang Y., You J., Yan J., Fu H., Zhang D. (2015). Continuous liquid level sensor based on a reflective long period fiber grating interferometer. Meas. Sci. Technol..

[17] Wang J., Luo C. (2012). Long-period fiber grating sensors for the measurement of liquid level and fluid-flow velocity. Sensors.

[18] Huang Y., Chen B., Chen G., Xiao H., Khan S.U. (2013). Simultaneous detection of liquid level and refractive index with a long-period fiber grating based sensor device. Meas. Sci. Technol..

[19] Yu Y., Hung H., Liaw S., Shih M., Kishikawa H., Goto N. (2017). Simultaneously two-parameter measurement using tilted fiber grating and long period fiber grating. Microw. Opt. Technol. Lett..

[20] Jiang M., Zhao Z., Li K., Yang F. (2018). Long-period fiber grating cascaded to thin-core fiber for simultaneous measurement of liquid refractive-index and temperature. Sens. Rev..

[21] First-Sensor.com Measuring Transition Points in Liquids with Different Densities with CLC and CLW Capacitive Level Sensors. https://www.yumpu.com/en/document/view/43889645/measuring-transition-points-in-liquids-with-different-sensortechnics.

[22] Huang Y., Xiao H. Detecting layered liquids using long period fiber grating sensors. Proceedings of the 2nd International Electronic Conference on Sensors and Applications.

[23] Li Y.J., Wei T., Montoya J.A., Saini S.V., Lan X.W., Tang X.L., Dong J.H., Xiao H. (2008). Measurement of CO_2_ laser irradiation induced refractive index modulation in single mode fiber toward long-period fiber grating design and fabrication. Appl. Opt..

[24] Shu X.W., Zhang L., Bennion I. (2002). Sensitivity characteristics of long period fiber gratings. J. Lightw. Technol..

[25] Yaws C.L. (1999). Chemical Properties Handbook.

[26] O’Neil M.J. (2006). The Merck Index: An Encyclopedia of Chemicals, Drugs, and Biologicals.

[27] Ivanov O.V., Nikitov S.A., Gulyaev Y.V. (2006). Cladding modes of optical fibers: Properties and applications. Physics.

[28] Patrick H.J., Kersey A.D., Bucholtz F. (1998). Analysis of the response of long period fiber gratings to external index of refraction. J. Lightwave Technol..

